# Countering the Identified Barriers to Delivered Oral Care for Children With Special Healthcare Needs: A Narrative Review

**DOI:** 10.7759/cureus.67561

**Published:** 2024-08-23

**Authors:** Hamdan Alamri

**Affiliations:** 1 Department of Preventive Dental Sciences, College of Dentistry, Majmaah University, Majmaah, SAU

**Keywords:** dental care for disabled oral hygiene, access to health care, dental care barriers, dental care, disabled children

## Abstract

Children with Special Health Care Needs (SHCNs) have poor oral health as a result of structural and systemic barriers. These children frequently have limited access to dental treatment, a higher prevalence and incidence of dental problems, and worse oral hygiene than the rest of the population. This review aims to offer an understanding of the existing oral care barriers of children with SHCNs. We reviewed the literature on children with SHCNs and their caregivers to identify the implications of the barriers faced by these individuals. Some of the perceived barriers to appropriate oral healthcare faced by these children include obstacles to adequate oral care and hygiene in the home, challenging behaviours, and limited preventive care and accessibility. We focussed on interventions and different management approaches to support the stakeholders responsible for these children. There is a need for strong communication as well as care coordination between caregivers, dentists, and other providers to achieve positive outcomes. The current dental healthcare system appears to desert the needed demands of this demographic.

## Introduction and background

Previous research has shown that the incidence of dental decay in children with special health care needs (SHCNs) is comparable to other children in the same age group. Nevertheless, the dental condition of children with SHCNs seems to decline much greater than that of the general people as they age. Children with SHCNs have fewer dental fillings, more missing teeth, and higher rates of untreated tooth decay than people in general [1, 2, 3, 4]. Furthermore, a comprehensive study conducted by Anders and Davis (2010) found that individuals with SHCNs have a greater frequency of untreated dental decays and periodontal disease as well than people in general [3]. 

There are serious repercussions from poor oral health, with some research showing how it affects children with SHCNs' general health [5]. Recent studies reveal that there are already health inequities for children with SHCNs. [6, 7, 8]. Presumptions concerning the oral health condition of these children, when examined based on the current research, are difficult to make, with a few studies showing that the dental health status of children with SHCNs is the same as that of children without needs. A few studies even indicate that children with SHCNs have better oral health than those without needs [9]. However, improvements in these children's health conditions may be attributed to increased oral health promotion and increased knowledge of the necessity of dental care and oral health among this demographic. 

Although barriers and accessibility to oral healthcare services have been widely investigated, there has been relatively little study on the oral health care challenges faced by children with SHCN. The aim of this narrative review is to identify as well as review some of the existing evidence regarding the existing oral health care barriers for children with SHCNs. A discussion of the applicability of different interventions to prevent and treat dental diseases is also presented, alongside some of the evidence-based strategies to promote oral health for Children with SHCNs.

## Review

Methods

We reviewed the existing literature about oral health for children with SHCNs to identify some of the barriers to oral healthcare faced by these. A literature search by the reviewer for relevant articles written in English in the MEDLINE Ovid database pertaining to the oral health of children with SHCNs, including preventive and treatment interventions, behavioural issues, and different managerial considerations. Published manuscripts were searched using the search terms “Dental Care for Disabled”, “Toothbrushing”, “Oral Hygiene”, “Health Promotion” “Patient Care Management” “and “Guidelines as Topic”. The researchers focussed on different management approaches as well as interventions to support these children. Strategies to support oral care providers in delivering optimal oral care to these children were also explored.

Result

A wide range of barriers was identified that ranged from physical limitations, communication behavioural challenges, sensory sensitivities, lack of awareness and insufficient information of families of children with SHCNs, and the lack of availability of specialised oral healthcare services and providers. Furthermore, the inability of children with SHCNs to self-care, in addition to limited access to professional services, makes overcoming these barriers especially important. 

Identified barriers

Behavioural challenges in children with SHCNs were amongst among the most commonly raised concerns in the literature. When children with SHCNs become apprehensive and noncompliant during home care, they may exhibit anxiety symptoms such as temper tantrums and emotional outbursts. These actions can be caused by the child's irritation as a consequence of the interruption to their regular routine or by them feeling uncomfortable in a new place, such as a dental care facility. In more acute situations, their violent attitude sometimes manifests as striking, biting, scraping, and even damaging belongings near them. The intensity of such behaviours can be caused by a gap in the type of demand and a lack of language development [10]. 

Numerous findings have shown that children with SHCNs experience significant levels of dread and anxiety [11, 12]. Research indicates that dental visits are correlated with fear, which may have an effect on oral health, particularly in children with SHCNs [13]. These children could get a bad impression of dental clinics as a result of this. In the waiting areas, negative responses are frequently noticed, which can lead to the cancellation of appointments before they've ever started. In the context of dentistry, challenging behaviours include self-harming activities, hypersensitivity to stimuli, hyperactivity, and a non-compliant demeanour [14]. Due to their hypersensitivity to tactile or auditory triggers, some children with SHCNs experience extreme stress in dental care facilities, which can lead to conditioned dread in children with autism [12]. Furthermore, certain kids could find it challenging to remain still in the chair, necessitating the use of restraint and preventive stabilisation [15]. In order to provide proper dental care, a treatment under general anaesthesia can be necessary if children with SHCNs exhibit extremely unsettling behaviour.

Parents of children with SHCNs highlighted that it was challenging to find a dentist and to have access to a dental clinic that could meet the oral health needs of their children [16]. It should be mentioned that the absence of dentists with the necessary training or their reluctance to serve children with SHCNs may be the cause of the restricted access to dental treatment. Additionally, the way oral health care practitioners treat their patients or themselves may deter parents or other carers of children with SHCNs from receiving dental care [16]. Trying to locate a dental facility that will take their kid or can meet their needs is frequently a difficult scenario for parents and other carers of children with SHCNs. This can be frustrating for carers, particularly if the child has behavioural challenges or is unwilling to comply. 

One of the reported challenges identified in the research was the oral care professionals' incapacity to handle children with SHCNs. Several studies have demonstrated a shortage of qualified providers to provide oral care for children with SHCNs [17, 18]. Many studies have found a shortage of adequate professional training in managing the necessities of these children, which is seen as one of the primary explanations why, according to parents, oral care professionals failed to handle children with SHCNs [17, 19, 20]. Given the restricted access to specialized oral care facilities, some authors have noticed that some oral care practitioners find providing oral care for children with SHCNs to be overly tense and demanding [21]. Furthermore, some oral care providers are unwilling to visit or treat these children, whether due to perceived poor remuneration or scheduling constraints [7]. The shortage of training offered to practitioners while in dentistry school shows that practitioners are not completely equipped to satisfy such a demand due to limited theoretical knowledge [21]. 

The parents of the children are also potential barriers because treatment becomes both costly and time-consuming. The expense of oral care for parents of children with SHCNs can be a substantial barrier to receiving care, potentially causing financial pressure. High costs for specialised dental services, equipment, and continuous treatments may make it difficult for parents to provide adequate oral health care for their children [22]. In addition, when accessing a specific oral health service in their region, parents must be aware of its presence. As a result, addressing the financial pressure around care and raising the awareness of these children's carers or parents regarding the availability and accessibility of various dental treatment facilities may help to alleviate some of the barriers highlighted. Furthermore, offering conveniently available information about nearby dental clinics, such as an online dental directory with the names of all dental clinics that successfully treat children with SHCNs, may reduce delays in accessing oral treatment.

Promotion and prevention strategies

Health promotion refers to the process of allowing people to have greater control over various health factors, thereby enhancing their own health [23]. Oral health promotion has an important role to play in improving quality of life via achieving better oral health, especially for Children with SHCNs. There is a need for additional support and attention towards the oral care of Children with SHCNs since it is often related to other conditions and behavioural and emotional disorders. 

Children with SHCNs require more effective oral hygiene approaches and tasks because they may lack the cognitive abilities and motor dexterity needed to comprehend or practice efficient oral hygiene [3]. These children may also have other linked illnesses, such as behavioural issues, chronic medical diseases, anxiety, or sensory impairment, which may have an influence on their dental hygiene plan. In one research, parents of children with SHCNs recognised certain important concerns associated with their children's handicap, such as an urge to gag, a hypersensitive mouth, trouble using a toothbrush, and a failure to properly wash their mouths [24]. Furthermore, the parents of these children are frequently not adequately educated and are unaware of the significance of oral hygiene, consequently, they do not prioritise oral hygiene [25]. To overcome these obstacles, children with SHCNs need reinforcement and assistance for oral hygiene from both oral care providers and carers [26].

Oral hygiene 

Promotion of Oral Care Habits

Children with SHCNs are at a high risk of developing dental caries. An overwhelming body of evidence indicates that fluoride is effective in caries control among children and adolescents [27, 28, 29]. There are various carriers to provide fluoride to children with SHCNs such as toothpaste, dietary additives, varnish, and mouth rinse. Fluoridated toothpastes are easily available and accessible and have been recommended for more than half a century. Moreover, approximately 95% of toothpastes that are available worldwide contain fluoride [30]. One of the benefits is that it does not require a lot of investment and parents should be instructed by oral healthcare providers about how to select a fluoride-containing toothpaste to address the child’s need.

Caregivers of children with SHCNs should actively work to prevent dental problems by encouraging good oral hygiene practices. Toothbrushing with fluoridated toothpaste on a regular basis helps prevent periodontal disease and dental caries in the general population [31, 32]. However, it is important to note that many research studies on oral health exclude individuals with SHCNs [33]. In addition, a Cochrane review showed that electric toothbrushes were an effective means of controlling gingivitis among the general population as compared to manual toothbrushes [34]. However, the benefits of the use of electric toothbrushes on Children with SHCNs have not been confirmed. 

Chlorhexidine rinse is one of the preventive measures known to control gingival inflammation. Children with SHCNs have more severe gingival inflammation as compared to the general population [35]. It was reported in a high-quality evidence that mild gingival inflammation is effectively reduced through the use of chlorhexidine for four to six weeks, while not enough evidence is available to understand if the chlorhexidine was effective in controlling gingivitis among individuals with moderate to severe inflammation [36]. However, the evidence for individuals with SHCNs is limited and the effectiveness of chlorohexidine might be compromised among this group. Further investigation should explore this area and whether it is an effective intervention to use at home for children with SHCNs with periodontal inflammation [37]. 

Some children with SHCNs have strong dislikes for toothbrushing and toothpaste and might consume a very restricted diet. Furthermore, some children with SHCNs are oversensitive to flavours, odours, and feels, which might be hurdles to accepting fluoride treatment when using toothpaste to clean their teeth. As a result, certain parents may choose to limit or eliminate the use of fluoride by denying topical fluoride therapy for their children, using fluoride-free toothpastes, etc. If toothpaste is not an option, examine alternative methods and approaches for administering fluoride with your oral care provider. It is thus extremely necessary that knowledge on the relevance of fluoride, protective measures for dental problems, and home oral care be given to carers in a clear and efficient way. This will allow children with SHCNs to receive adequate fluoride exposure and aid in avoiding or decreasing dental diseases.

While dental caries can be prevented using sealants and fluorides, tooth decay can be prevented through diet. The growth of oral bacteria is fostered by highly refined carbohydrates and sugars [38]. Many children are hooked to sugar, and children with SHCNs frequently lack the cognitive capacity to control their consumption. Children with emotional and learning impairments frequently want diets rich in sugar and carbs, which can cause dental problems [39]. Children at high risk of dental decays, such as SHCNs, require additional involvement from parents and carers in lowering sugar intake since this will affect their oral health [21, 40]. This is particularly relevant given the Saudi population's high intake of sugary foods and beverages [41]. A non-cariogenic diet can be used to refrain from oral disease over time. Dietary advice should be provided to these youngsters as part of public oral health campaigns from an early age. Parents and dental care practitioners should collaborate to reduce the risk of caries by tracking the frequency with which cariogenic foods and beverages are consumed, as well as boosting professional and self-care protective measures. The oral adverse effects of any drugs should also be considered, and sugar-free treatments should be utilised wherever feasible [42].

Oral Health Education Programmes

Research suggests the oral health behaviours, attitudes, and knowledge of carers of children with SHCNs might either support or hinder dental care or dental health-promoting activities in their children [29]. Cultivating understanding among carers and children with SHCNs may have a favourable influence on their oral health outcomes. As a result, dental practitioners must develop specific training and education programmes for carers and children with SHCNs, focussing on prevalent issues that parents and children experience. Furthermore, oral health education programs intended to strengthen and sustain positive behaviours among these children, as well as to introduce new, essential behaviours such as more frequent dental visits, should be implemented to enhance their oral health. The notion of encouragement, as well as the repeating of oral hygiene instructions, has considerable immediate and long-term benefits [43]. 

Oral health programmes should help overcome the perceived barriers equipping caregivers with the knowledge to maintain the oral hygiene of their child. The results from a recent review clearly emphasise the need to explore education programmes about oral health for this demographic [8]. Community-based fluoridation strategies are among the most effective and equitable methods for the control of dental caries in children [44]. Some studies have reported that through school-based programmes individuals with learning disabilities had the potential to improve their plaque removal efficacy in the short term [37, 45]. Furthermore, training carers to brush the teeth of individuals with disabilities may improve carers' oral hygiene knowledge [37]. Community-based oral health interventions combined with both supervised toothbrushing and oral health education were reported to reduce dental caries in children [46]. Moreover, the delivery of preventive treatments such as fluoride varnish and pit and fissure sealant, alongside providing school-based dental screening programs can improve the oral health outcome of Children with SHCNs [47].

The lack of promotion of routine oral health visits should be addressed.. Bhaskar et al. (2014) conducted a systematic review regarding the importance of preventative dental visits for children. The authors concluded that preventative visits to dental clinics had a positive impact on reducing dental caries, specifically for children at high risk of dental caries, such as children with SHCNs [48]. Another study concluded that those who missed appointments had four times the risk of caries [49].

The dental home

There is a need to design approaches to preventive oral hygiene for the convenience of children with SHCNs to help overcome some of the reported barriers. One way to help these children foster a lifetime of oral health good practices is by assisting them in developing healthy attitudes and good oral hygiene practices from childhood [50]. It is important to involve the parents of the child with SHCNs early in life to promote lifelong habits [51]. Parents that are well-informed, educated as well as supported are more capable of delivering proper oral care for their child at home and offering a great foundation for the child [52]. A policy on dental homes was developed by The American Academy of Paediatric Dentistry (AAPD) and revised in 2018. The AAPD describes a dental home as an “ongoing relationship between the dentist and the patient, inclusive of all aspects of oral health care delivered in a comprehensive, continuously accessible, coordinated, and family-centered way” [53]. 

The concept of a dental home follows directly from the definition of a medical home as a place where children and families obtain “accessible, continuous, comprehensive, family centred, coordinated, compassionate, and culturally effective medical care” [54]. One of the key benefits of this approach is that a dental home set up early in life may minimise or prevent dental issues and reduce the fear or anxiety of children when receiving oral care treatment. It has been recognised that a key aspect of both medical and dental homes is ‘care coordination’ among dental and medical providers, as a method for detecting as well as intervening to prevent dental issues [55] However, the current literature seems to offer limited comprehension of the concept and future research is needed to address its limitations and applicability in paediatric dentistry and especially for Children with SHCNs [56].

Perceived issues in professional care

Preventative Measures 

Fluoride varnish has a substantial caries-inhibiting effect and is one of the best methods available of topical fluoride application [29]. There is a high-quality evidence available on the usefulness and efficiency of fluoride varnish in preventing caries in both primary as well as permanent dentitions [28]. Various systematic reviews confirm that applying topical fluoride twice or more a year results in a mean reduction of caries increment of around 45% in permanent dentition and 40% in primary dentition. The research supports the view that existing lesions can also benefit from the application of varnish on the roots of permanent teeth and the smooth surfaces of primary teeth. Most evidence of efficacy is based on the application of sodium fluoride 22,600 ppm varnish [29].

Silver diamine fluoride (SDF) is an arresting treatment approach for carious lesions such as exposed fissures and root surfaces and serves as a low-cost method for preventing active dental decay [57]. This approach can offer great help for children, especially in cases where a lack of cooperation and challenging behaviour limits habitual oral care [58]. SDF contains ~5% fluoride, which is twice as much as in varnish. Its high fluoride composition facilitates more effective diffusion into dentin and enamel with staining as its only known side effect [58]. Additional delivery approaches, such as fluoride rinses, foams, gels, and high-fluoride toothpastes, have been developed to maximise the prevented fraction of caries lesions and minimise the application frequency and duration of fluoride [38]. All of these interventions need to be age-appropriate and should be helpful for clinicians to assess and use, fully conscious of the side effects of fluoride when delivering care for these Children with SHCNs. However, the success of these treatments depends on the child’s compliance and the clinician’s ability to manage Children with SHCNs in dental clinics [59]. 

Pit-and-fissure sealants, such as resin sealants, are considered the standard of care for the prevention of non-cavitated lesions on occlusal surfaces [28]. Mechanical barriers such as pit-and-fissure sealants might benefit Children with SHCNs and reduce the risk of developing caries. This group of children is a priority for the use of fissure sealants [40]. In addition, sealant over carious lesions as in the Hall technique, where a preformed stainless steel or crown and luting cement is placed over the tooth, is one of the most effective caries lesion treatments in primary teeth and might benefit these children [60]. This technique is simpler and less invasive than the conventional restorative approach, which can be more acceptable to Children with SHCNs.

Training and Expertise of Dentists

Educational barriers can impact the abilities of oral care provider to deliver efficient and effective care to Children with SHCNs. Behavioural issues, in addition to complicated medical and physical circumstances, can make it difficult for oral care professionals who lack knowledge and competence to offer the necessary therapy. As a result, some practitioners may avoid treating people with SHCNs or react with boredom and irritation since it takes more work and time [61]. If the dental staff is not properly trained, problems are more likely to occur, resulting in insufficient treatment. 

Meanwhile, the dentist is another potential barrier, due to their inadequate knowledge and experiences [21]. One study reports that dentists prefer not to treat patients who may be problematic, despite The Disability Discrimination Act stating that it is illegal for the oral health provider to treat children with special needs less favourably [62]. It was observed by Casamassimo et al. (2004) that an oral care provider who was properly trained and had received additional training would usually take on the oral care for CSHCN without any reservations and was also less likely to see the level of mental illness or disability or behaviour of the child as a challenge in providing proper dental treatment. Furthermore, a higher tolerance and acceptance rate in treating these children were found among oral care providers who had clinical experience and theoretical information in this area [21]. 

Creating an enjoyable setting to foster lasting relationships between patients and clinicians in order to enhance oral health is an essential responsibility for dentists working with this age group [14]. limited communication between clinicians and children has been identified as a possible source of dental fear during care [63, 64]. While caring for children with SHCNs, an acceptable method of communication should be created throughout each visit. There must be attempts to properly connect with the patient while providing treatment. The kid's parents or carer may be asked to support communication and supply extra information that the youngster is unable to communicate [65].

Communication is crucial, but oral healthcare providers must not consider communication solely to be a way of receiving or transferring information. Communication must be undertaken to develop a human connection so that the preferences, needs, and beliefs of the patient are understood. This important finding highlights that dental healthcare practitioners must put the patients first and provide holistic care to them [56]. 

Management of children with SHCNs in a dental setting

Many studies have addressed the problems of providing sufficient oral care to children with SHCNs [66]. Challenges in managing children with SHCNs stem from low tolerance, behavioural challenges, a lack of collaboration, the oral care provider's inexperience, and a lack of appropriate assistance from carers during care. Children with SHCNs are a diverse population with varying levels of requirements. Some of them may be able to cooperate with dental care using basic behavioural strategies, especially if the oral care practitioner is willing, skilled, and knowledgeable enough to offer the necessary treatment [67].

Past medical or oral experiences can negatively impact a child's cooperation and ability to cope with dental treatment [68]. To create a comfortable environment, children with SHCNs should be taken to a clinic for a familiarisation visit with the dentist and staff [69]. Minimizing olfactory, auditory, and visual stimuli, and keeping coping objects within reach, is crucial. The process should be gradual [70].

Children with SHCNs often face challenges in behaviour management, as they display resistant behaviours that can interfere with safe dental care [71]. Parents and caregivers are essential for managing unexpected behaviour in the dental room [72]. Physical stabilisation is considered essential for children with inadequate attitude guidance, but it should be limited and only after acclimatization, distraction, and desensitization strategies have proven ineffective [73, 74]. If stabilization is not feasible, sedation can be an alternative behavioural management technique [75].

Oral healthcare providers use various techniques for behavioral modification and pharmacological control strategies, but children with greater needs or disabilities may require general anaesthesia or adjunctive sedation [76]. A study found that a 50% mixture of nitrous oxide/oxygen was successful in 92% of cases, with minor side effects [77]. Children with SHCNs are frequently treated with general anaesthesia, but it should be the last resort due to adverse drug interactions, lingering processes, and high costs, potentially reducing dental visit frequency [78,79]. When general anaesthesia is necessary, the dental profession should discuss all other options with caregivers to ensure they understand that this is a last resort for the child's benefit, not for the dentist's convenience. This ensures a clear understanding of the decision-making process.

The potential benefit of using cutting-edge technology such as artificial intelligence, virtual reality, teledentistry, augmented reality, and other breakthroughs in dental care for children with SHCNs remains a subject of little focus of scientific research. However, there is a positive perspective that these revolutionary technologies will alter the way dental care is offered to children with SHCNs, perhaps providing more customised, accessible, and effective treatments. These innovations have the potential to improve treatment outcomes, patient experiences, and the provision of specialised care that is critical for maintaining good dental health and general well-being in children with disabilities [80, 81]. More study and inquiry are required to properly understand the scope of these advantages in the context of oral health care for this particular demographic.

Understanding a patient's medical history is crucial for dental treatment, as it helps practitioners understand risks and deliver appropriate care. Children with sensory issues, such as those with SHCNs, often require a thorough medical history to design primary care [72]. Dentists should record the child's health conditions and diseases at every visit, and if the patient cannot provide the necessary information, consultation with parents may be necessary. If the child's needs are beyond the dentist's skills, a referral should be made to the appropriate practitioner [72, 82].

The timing of appointments is crucial for children with special healthcare needs. The initial contact allows for addressing basic oral care requirements and determining the effectiveness of a dentist. Staff members should identify SHCNs and determine the need for longer appointments [83]. Oral care providers should consult with other medical teams for optimal treatment and be prepared to handle medical emergencies.

There are a few limitations that must be acknowledged while performing this narrative review. These include the potential for bias in study selection, the inherent subjectivity that can affect study selection and interpretation, a lack of a structured review, a systematic approach that could result in incomplete coverage of the literature, and the difficulties in effectively synthesising and appraising diverse data sources. A systematic review would have to be conducted to accomplish these limitations. All these elements work together to influence the validity and breadth of the findings that may be made from this review.

## Conclusions

Future strategies for addressing the oral and dental health needs of children with SHCNs should concentrate on enhancing the child's cooperation in the dental clinic, expanding access to appropriate and professional dental care, and closely collaborating with the child's carers to create a personalised preventive program appropriate for the child's age and condition. Introducing basic adjustments to dental clinic logistics, such as incorporating children with SHCNs and their parents or carers as major partners in improving oral hygiene practice and preventative measures for these children, will provide the groundwork for a brighter future of oral healthcare. Whilst there is evidence of the effectiveness of some interventions, there needs to be further research to confirm their effectiveness for children with SHCNs. Further, efforts and interventions should be focused on encouraging parents in playing their role in preventative/routine visits to the dental clinic.
